# Whole-pattern fitting technique in serial femtosecond nanocrystallography

**DOI:** 10.1107/S2052252516001238

**Published:** 2016-02-12

**Authors:** Ruben A. Dilanian, Sophie R. Williams, Andrew V. Martin, Victor A. Streltsov, Harry M. Quiney

**Affiliations:** a ARC Centre of Excellence for Advanced Molecular Imaging, School of Physics, University of Melbourne, Parkville, Victoria 3010, Australia; b CSIRO Manufacturing Flagship, Parkville, Victoria 3052, Australia

**Keywords:** protein nanocrystallography, peak-shape analysis, whole-pattern fitting, X-ray free-electron lasers, nanocrystals, SFX, protein structure, XFEL

## Abstract

Serial femtosecond X-ray crystallography (SFX) has created new opportunities in the field of structural analysis of protein nanocrystals. The intensity and timescale characteristics of the X-ray free-electron laser sources used in SFX experiments necessitate the analysis of a large collection of individual crystals of variable shape and quality to ultimately solve a single, average crystal structure. Ensembles of crystals are commonly encountered in powder diffraction, but serial crystallography is different because each crystal is measured individually and can be oriented *via* indexing and merged into a three-dimensional data set, as is done for conventional crystallography data. In this way, serial femtosecond crystallography data lie in between conventional crystallography data and powder diffraction data, sharing features of both. The extremely small sizes of nanocrystals, as well as the possible imperfections of their crystallite structure, significantly affect the diffraction pattern and raise the question of how best to extract accurate structure-factor moduli from serial crystallography data. Here it is demonstrated that whole-pattern fitting techniques established for one-dimensional powder diffraction analysis can be feasibly extended to higher dimensions for the analysis of merged SFX diffraction data. It is shown that for very small crystals, whole-pattern fitting methods are more accurate than Monte Carlo integration methods that are currently used.

## Abstract 2

Extracting structure-factor moduli from diffraction patterns of protein nanocrystals is one of the critical issues of serial femtosecond X-ray crystallography. Unlike a conventional crystallography experiment, serial femtosecond crystallography combines data from hundreds or thousands of crystals of varying size and quality, a situation reminiscent of powder diffraction. Here the whole-pattern fitting technique, originally designed for one-dimensional powder diffraction crystallography, has been reconsidered and applied to the analysis of higher-dimensional serial femtosecond X-ray crystallography data. For nanocrystals with a small number of unit cells, the whole-pattern fitting approach is shown to be more accurate than integration-based Monte Carlo methods.

## 1. Introduction   

Serial femtosecond X-ray crystallography (SFX) (Chapman *et al.*, 2011 ▸; Spence *et al.*, 2012 ▸), which emerged with the commissioning of hard X-ray free-electron laser (XFEL) sources, provides a unique opportunity for modern biology to conduct structural analysis of proteins which have previously been inaccessible to study because of the extremely small size of crystals that they form (*e.g.* submicron). The capabilities of the SFX approach have been successfully tested in recent studies of submicron and nanoscale protein crystals (Chapman *et al.*, 2011 ▸; Boutet *et al.*, 2012 ▸; Johansson *et al.*, 2012 ▸, 2013 ▸; Koopmann *et al.*, 2012 ▸; Redecke *et al.*, 2013 ▸; Aquila *et al.*, 2012 ▸; Liu *et al.*, 2013 ▸; Demirci *et al.*, 2013 ▸; Kupitz *et al.*, 2014 ▸).

The SFX technique involves illuminating a stream of randomly oriented protein crystals of various sizes and orientations by an extremely bright and ultra-short (tens or hundreds of femtoseconds) XFEL source and properly merging the obtained diffraction data. SFX experiments are performed in this manner due to the destructive nature of the XFEL source for which a single exposure can be expected to cause the disintegration of a nanocrystal (Neutze *et al.*, 2000 ▸). Diffraction information is obtained from different orientations of the reciprocal lattice of the target structure through continual replenishment of individual crystal samples in the XFEL beam. Whilst some characteristics of single crystals can be obtained from processing individual SFX diffraction patterns, the solution of a three-dimensional crystal structure requires the processing of large numbers of SFX diffraction patterns. The solution obtained is then an average crystal unit-cell structure found from data that are intrinsically based on distributions of both crystal sizes and qualities.

The SFX approach has a number of problems that have not been so critical to protein crystallography until now. In an SFX experiment, a single crystal effectively stands still during X-ray illumination due to the femtosecond timescale of an XFEL pulse. A collected diffraction pattern may then be thought of as a diffraction ‘snapshot’ that represents the diffraction of a single XFEL pulse from a single particle of finite size and a unique orientation. Furthermore, the small size of the protein crystals illuminated by the XFEL source can create broad intensity distributions around Bragg reflections (Yefanov *et al.*, 2014 ▸). Since there is no time for any rotations of the sample during the XFEL pulse, only partial information about the crystal shape transform is recorded on the diffraction pattern. Consequently, the shapes of the observed Bragg reflections may vary significantly from shot to shot and within a single image (White *et al.*, 2012 ▸). The SFX data set consists of two-dimensional diffraction patterns comprising partially recorded information from different crystals. To resolve these issues and to obtain the structure-factor moduli of Bragg reflections, which contain encoded molecular structural information, the SFX approach has relied on the Monte Carlo integration method (Kirian *et al.*, 2010 ▸, 2011 ▸; White *et al.*, 2012 ▸; White, 2014 ▸), in which a large number of diffraction patterns are separately analysed. For each diffraction pattern, the recorded intensities are summed within a fixed integration volume around each Bragg reflection and the obtained integrated intensities are averaged over all ‘snapshots’. The choice of the integration area is critical for the integration method to work and the accuracy of this procedure determines the accuracy of further structural analysis. Current crystallographic programs, such as *MOSFLM* (Leslie & Powell, 2007 ▸), *CrystFEL* (White *et al.*, 2012 ▸) or *Cheetah* (Barty *et al.*, 2014 ▸), use either circular or rectangular integration areas for the analysis. It has also been suggested that proper integration of diffracted intensities within the Wigner–Seitz cell around each Bragg reflection could be used (Kirian *et al.*, 2010 ▸, 2011 ▸).

The approaches currently used in protein crystallography to extract structure-factor moduli from the diffraction pattern (Leslie & Powell, 2007 ▸; Kabsch, 2010 ▸; Kirian *et al.*, 2010 ▸; White *et al.*, 2012 ▸; Barty *et al.*, 2014 ▸) rely on the segregation method, *i.e.* the diffraction pattern is considered as a discrete set of completely isolated Bragg reflections. In this case, the intensity distribution within a predefined region around a given Bragg reflection is used to extract structure-factor moduli. Here we present a whole-pattern fitting technique that uses a continuous description of the merged diffraction data. This holds some similarities with profile-fitting methods used for single-crystal data, such as *MOSFLM* (Leslie & Powell, 2007 ▸) and *XDS* (Kabsch, 2010 ▸), and those recently introduced for SFX data analysis, such as *nXDS* (Kabsch, 2014 ▸). A key difference is that the latter approaches rely on the segregation method for the extraction of structure-factor moduli with the assumption that Bragg reflections can be separated and integrated. These approaches are based on the scaling of learned peak shapes found from strong peaks that are assumed to be isolated, rather than using analytical expressions to model a continuous intensity distribution. Moreover, the integration approach relies on the statement that the resulting shapes of Bragg reflections on the SFX diffraction pattern are governed by the averaged crystal shape transform only (Kirian *et al.*, 2010 ▸; White *et al.*, 2012 ▸), which is the same for all Bragg reflections. Thus, assuming a defect-free structure of protein nanocrystals, the structure-factor moduli can be extracted from SFX data by the integration of diffracted intensities within a predefined region around all Bragg reflections. We will later refer to this as the ‘integration approach’.

We have recently shown (Dilanian *et al.*, 2013 ▸), however, that the size and the quality of individual protein nanocrystals illuminated by an X-ray source during the SFX experiment significantly affect the resulting SFX diffraction pattern. Given the asymptotic behaviours of the Bragg reflections due to the size distribution of the nanocrystals and the scattering in inter-Bragg regions due to the size of individual crystals, the assumption that the Bragg reflections are isolated cannot always be satisfied. The smaller the nanocrystals and the bigger the unit-cell parameters of the protein crystal, the stronger is the influence of asymptotes of the nearest Bragg reflections on the intensity distribution of a given Bragg reflection. Moreover, the large surface-to-volume ratio of protein nanocrystals leads to significant contributions from surface effects, such as structural disorder, impurities and lattice distortions near the surface of the crystal *etc*. (Feher & Kam, 1985 ▸; Grant & Saville, 1994 ▸; Caylor *et al.*, 1999 ▸; Malkin & Thorne, 2004 ▸), to the diffraction pattern. In such cases the shape of Bragg reflections will not be exclusively governed by the averaged crystal shape transform and, therefore, will not be identical for all reflections, varying with the scattering vector (Dilanian *et al.*, 2013 ▸). Consequently, restriction of the integration areas may lead to an incorrect estimation of the corresponding structure-factor moduli.

In this paper, we present an approach to the extraction of the structure-factor moduli of Bragg reflections from SFX data which resolves the issue mentioned above. A key idea in this approach is the treatment of the merged SFX diffraction data set as a continuous function of the scattering vector, **q**, and not as a discrete set of Bragg reflections. Such a treatment is demonstrated in the three-dimensional merging of whole two-dimensional SFX diffraction patterns by Yefanov *et al.* (2014 ▸). This involves the mapping of whole two-dimensional diffraction patterns (according to the orientations of individual particles) to three-dimensional **q** space for further analysis. The result is a diffraction data volume comprised of an ensemble of particles of different sizes and structural qualities that retains the dimensionality of the reciprocal crystal lattice. As indicated by Yefanov *et al.*, it is possible to take into account fluctuations in the incident pulse intensity and beam convergence during an SFX experiment by weighting intensities during merging based on single-shot spectra. From this point of view, there is a similarity in the formation of a merged SFX diffraction data set and a powder diffraction pattern, where the shapes of Bragg reflections are similarly formed by a collection of independent scatterers of varying characteristics. Differences exist in that individual SFX diffraction patterns may be first processed before merging and that the merged data need not be collapsed into a function of the diffracted intensity with respect to the scattering vector magnitude (*i.e.* in one dimension) due to the ability to estimate the orientation of individual crystals. Instead, two-dimensional or three-dimensional merged SFX diffraction data sets can be formed. For a sufficient number of independently scattered particles, the shapes of the resulting Bragg reflections in the merged SFX diffraction data volume, as well as the asymptotic behaviours of their tails, will be governed by the statistical properties of distribution functions, which are characterized by the variations of particle size and structural imperfections (Suortti & Jennings, 1977 ▸; Suortti *et al.*, 1979 ▸; Young & Wiles, 1982 ▸), and not by properties of individual crystallites. Consequently, the structure-factor moduli may be extracted from the merged SFX data by fitting the whole SFX diffraction pattern using analytical peak-shape functions, which are defined over the entire range of **q** space, rather than only within restricted areas around individual Bragg reflections. Moreover, the fitting approach allows us to incorporate characteristics of Bragg reflections and the scattering in inter-Bragg regions into the analysis *via* the adjustable parameters of the peak-shape function, such as unit-cell parameters, width of the Bragg reflection or asymmetry of the reflection. Here, we demonstrate that the fitting approach may provide more accurate and robust results in extraction of structure-factor moduli from protein nanocrystals compared to the integration approaches developed so far.

## 2. General considerations   

We base our analysis on the consideration of finite crystals and on the assumption that the merged SFX diffraction pattern collected from a stream of nanoscale protein crystals of various sizes and qualities is a continuous function of the scattering vector, **q**. We start with the assumption that the scattering factor for a finite crystal can be expressed as
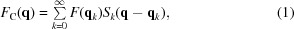
where 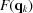 is the structure factor, 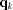 is the Bragg position of the *k*th Bragg reflection and 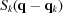 is a series of functions centred at Bragg positions, which arises from the finite crystal lattice. We will refer to 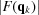 as the structure-factor modulus of the *k*th Bragg reflection here. Equation (1) is appropriate for crystals with whole unit cells (Ino & Minami, 1979 ▸) and can account for lattice imperfections like strain or lattice defects. For a perfect finite lattice, 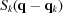 is identical for all values of *k*, but in general it can vary for different Bragg peaks. We note that in the nanocrystallography literature (Kirian *et al.*, 2010 ▸, 2014 ▸; Spence *et al.*, 2011 ▸), the scattering factor for a finite crystal is commonly expressed as a product of continuous unit-cell and lattice scattering factors, which for an ideal crystal with whole unit cells can be written in the form of equation (1) (see Appendix *A* [App appa]). Equation (1) cannot describe models of the crystal surface that prevent the continuous scattering factor of the unit cell, 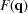, from being completely characterized by discrete samples of 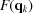 (Ino & Minami, 1979 ▸), such as the presence of incomplete unit cells on the crystal surface.

Assuming the validity of equation (1), the diffracted X-ray intensity distribution is given by 
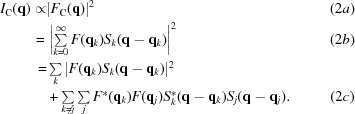
The second summation term in equation (2*c*) is typically neglected (James, 1954 ▸; Hosemann & Bagchi, 1962 ▸; Guinier, 1963 ▸; Ino & Minami, 1979 ▸). For sufficiently large crystals, equation (2*c*) is well approximated by the first summation term solely (James, 1954 ▸; Guinier, 1963 ▸). If the crystal surface is modelled by a random shift of the lattice with respect to the crystal’s centre, Ino & Minami (1979 ▸) show that the second summation can also disappear for small crystals. We note, however, that this model also requires a continuous dependence on the scattering factor, 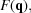 and is not consistent with the underlying assumptions made within equation (1), such as the presence of whole unit cells. In fact, the second summation contains phase information that is targeted by new methods for directly phasing SFX data (Spence *et al.*, 2011 ▸). In light of the progress in the direct phasing of finite crystals (Elser, 2013 ▸; Liu *et al.*, 2014 ▸; Kirian *et al.*, 2014 ▸, 2015 ▸), it is unlikely that the second summation is entirely absent for SFX data. Here, however, our goal is to obtain an improved estimate of the structure-factor moduli 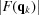 by accurately modelling the first summation of equation (2*c*) and not to pursue the additional phase information contained in the second summation term. We do so without invoking the physical assumptions of Ino & Minami (1979 ▸) of random shifts. We test the validity of ignoring the second term *via* simulation in §4. Further work may consider the contribution of this term more rigorously.

The model used here for the three-dimensional diffracted intensity distribution, merged from a collection of *N* finite crystals, can then be written as 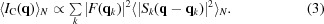
This holds similarities to the one-dimensional intensity distribution used in the profile-fitting analysis of powder diffraction data (Rietveld, 1967 ▸), which can be given by
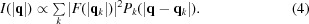
The above expression represents a whole-pattern fitting scheme in which a set of sampling points within a diffraction pattern, 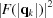, and an associated set of peak profile functions, 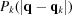, are chosen to evaluate the continuous function, 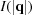. Based on the aforementioned similarities of SFX and powder diffraction data, we propose that the average shape-transform function contained in our model, 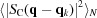, may also be modelled by continuous peak-shape functions, 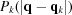. For the proposed whole-pattern fitting analysis, we define the modelled intensity distribution of the merged SFX diffraction as
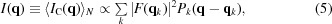
where the summation is performed over all Bragg reflections, contributing to the intensity distribution, 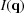, for a given scattering vector, **q**. 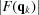 is the structure-factor modulus of the *k*th Bragg reflection, which we seek to accurately extract. It should be noted that in the modelled continuous intensity distributions [equations (3), (5)], all contributions from individual crystals are given equal weight.

With this model, each point of the resulting SFX diffraction pattern is affected by all Bragg reflections *via* the corresponding peak-shape functions, 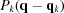. The intensity variation between neighbouring Bragg reflections will be governed by the collective contribution of both reflections. The degree of this ‘overlapping’ of the peak-shape functions depends on averaged size of the crystallites illuminated by X-rays, the size of the unit cell and the structural quality of protein crystals. From this point of view, Bragg reflections can be considered as isolated only if the corresponding peak-shape functions tend to zero within the Wigner–Seitz cell region around each Bragg reflection, which allows one to disregard the scattering in the inter-Bragg regions. In this case, the aforementioned segregation method provides correct estimations of the structure-factor moduli. In general, however, the collective contribution of Bragg reflections to the intensity distribution cannot be neglected and the scattering in inter-Bragg regions should be included in the analysis of the diffraction pattern (Dilanian *et al.*, 2013 ▸). Thus, consideration of the diffraction pattern as a continuous function of the scattering vector [equations (3), (5)], which forms by a collective contribution of all Bragg reflections *via* the corresponding peak-shape functions, may provide a more comprehensive approach for the analysis of diffraction data from a stream of nanoscale protein crystals.

The presence of whole unit cells has been assumed in the simulations presented here. The effects of partial unit cells present on the surface of protein nanocrystals for structural analysis have been considered by others (*e.g.* Liu *et al.*, 2014 ▸; Kirian *et al.*, 2014 ▸). Because of the finite size of nanocrystals, partial unit cells can influence intensity distributions between Bragg positions and introduce further ambiguities regarding the definition of the unit cell for a finite crystal. While the results of simulations presented here have only contained whole unit cells, we will consider the presence of partial unit cells in future work for which modifications may be introduced to the model used for the scattering factor for a finite crystal.

Two different schemes for extracting structure-factor moduli are considered here – an integration approach, based upon the consideration of discrete sets of Bragg reflections, and a whole-pattern fitting approach, based upon a continuous treatment of the diffraction data. The choice between these approaches depends on the average size of protein crystals, their size distribution and imperfections in crystal structure. According to our previous analysis, the consideration of the diffraction pattern as a continuous function of the scattering vector is crucial when the fraction of unit cells adjacent to the surface of the protein crystal exceeds 10% of the total number of unit cells. This suggests an effective size limit of 1 µm for a protein crystal with an average unit-cell parameter of 100 Å (Dilanian *et al.*, 2013 ▸).

Integration and whole-pattern fitting approaches are used here to extract structure-factor moduli from simulated diffraction patterns in order to evaluate the effectiveness and accuracy of both approaches in the analysis of SFX data from protein nanocrystals of various sizes. In the case of the fitting approach, equation (5), we extend whole-pattern fitting procedures developed for the analysis of powder diffraction data (Le Bail *et al.*, 1988 ▸) from fitting of one-dimensional intensity profiles to higher dimensions. Comparative analysis is presented in §4.

## 3. Fitting procedure   

The fitting procedure outlined here is conducted in a similar manner to that described by Le Bail *et al.* (1988 ▸). To begin, knowledge of the crystal structure (*i.e.* unit-cell parameters and space-group symmetry) and experimental geometry are used to predict Bragg peak locations in the modelled intensity distribution [equation (5)]. These positions can later be refined. Initial structure-factor moduli, 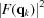, can be estimated by Wilson’s statistics (Giacovazzo, 2011 ▸). The initial modelled distribution [equation (5)] is calculated continuously using the estimates of structure-factor moduli and peak-shape distributions, based upon the chosen form of the peak-shape function and peak-shape parameters that are input by the user.

The peak-shape parameters are refined in each iteration of the whole-pattern fitting procedure by minimizing the error-cost function,
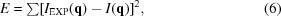
where 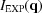 is the measured intensity and 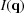 is the calculated continuous intensity distribution [equation (5)]. The summation is performed over all measured points of the diffraction pattern, rather than being assessed only at Bragg positions.

As in Le Bail analysis (Le Bail *et al.*, 1988 ▸), the structure-factor moduli are estimated at the conclusion of each parameter refinement cycle and are fed in to the next cycle as the input structure-factor moduli. We propose the estimation of the structure-factor moduli based upon the form of modelled intensity distribution [equation (5)]. This can be evaluated at Bragg locations to give
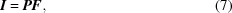
where ***I*** is a column matrix with elements, 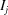, given by the observed peak intensity in the merged diffraction data set for the *j*th Bragg reflection and ***F*** is a column matrix with elements, 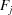, given by the squared structure-factor moduli, 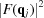. The square matrix, ***P***, has elements, 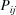, given by the contribution from the peak-shape distribution for the *i*th reflection relative to the position of the *j*th reflection, *i.e.* 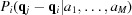. The 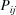 elements are evaluated based on the current peak-shape parameters, 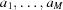. After each refinement cycle, the structure-factor moduli can then be estimated on the basis of the current peak shapes through inversion of the ***P*** matrix, using in our case the Gaussian elimination method, to find the ***F*** column matrix elements that satisfy equation (7) for the merged diffraction intensity distribution. In this manner, structure-factor moduli are iteratively estimated at the end of each fitting cycle.

The accuracy of the fitting process is monitored by two *R* factors, 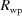 and 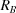. The standard definitions of these factors are used; see the comprehensive review by Hill & Fischer (1990 ▸). After a good fit between the modelled intensity distribution and merged intensity distribution is achieved, the current structure-factor moduli are extracted. Wilson statistics can then be employed to include scaling and thermal correction factors to the extracted structure-factor moduli (Giacovazzo, 2011 ▸).

### 3.1. Peak-shape function   

In the analysis presented here, we have chosen to model the peak shapes formed from the merged diffraction data with normalized pseudo-Voigt functions. This choice was motivated primarily by the pseudo-Voigt function’s widespread use in the analysis of powder diffraction data collected from crystal samples of varying size, shape and quality (*e.g.* Young & Wiles, 1982 ▸; Langford, 1999 ▸) and by the function’s considerable flexibility in form. The modelling of peak shapes with analytical functions such as the pseudo-Voigt function also improves computational speed and efficiency. It should be noted, however, that the general scheme presented here is based on the broader form of the modelled intensity distribution expressed in equation (5), for which alternative peak-shape distributions could be readily incorporated. Improvements to this approach might be made with greater theoretical consideration of the expected form of peak-shape distributions.

In this case, the calculated intensity distribution of the *k*th Bragg reflection from equation (5) can be written as 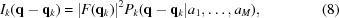
where 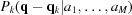 represents normalized pseudo-Voigt functions, 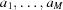 are adjustable peak-shape parameters, and *M* is the total number of adjustable parameters. In general, each of the adjustable parameters, 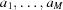, can be represented by an analytical function of the scattering vector, **q**, which takes into account possible variations of shapes of individual Bragg reflections with respect to the scattering vector.

We define an extended pseudo-Voigt peak-shape function in the following way:
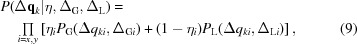
where 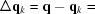 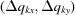. 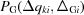 and 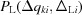 are normalized Gaussian and Lorentzian functions, respectively, with widths defined by 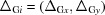 and 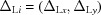. The parameters 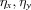 determine the weighting of both functions in the peak shape.

## 4. Results and discussion   

In this section we show the results of the extraction of the structure-factor moduli of the Bragg reflections from merged two-dimensional diffraction patterns using the whole-pattern fitting approach. The following is presented to demonstrate the feasibility of the extension of established powder diffraction analysis techniques to higher-dimensional SFX diffraction data. While the simulated diffraction data correspond to two-dimensional crystallographic planes, we expect this technique to be readily extendable to three-dimensional diffraction data.

### 4.1. Simulations   

The sugar-binding domain of langerin protein with the F241L mutation (Chabrol *et al.*, 2015 ▸; PDB entry 4AK8) was used as a test case for simulation studies. This protein has *P*4_2_ space-group symmetry and unit-cell parameters of *a* = *b* = 79.959, *c* = 90.419 Å. Simulations were performed to test the applicability of the whole-pattern fitting method to data merged from crystals of varying mean size. This was investigated by simulating several sets of needle-like nanocrystals of langerin with the numbers of unit cells in each crystal randomly generated from log-normal distributions. Both the shape and size of individual crystals were allowed to vary by sampling from independent size distributions that were taken to correspond to the orthogonal dimensions of a crystal. Finite-lattice transforms [see equations (16) and (17) in the Appendix and Dilanian *et al.* (2013 ▸), for details] were calculated along the [100] and [010] directions of the reciprocal crystal lattice.

The mean numbers of unit cells were varied between sets of simulated crystals as follows. The mean number of unit cells in the *Y* direction, 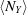, was varied from 60 unit cells progressively down to 30 unit cells in increments of 5, while the mean number of unit cells in the *X* direction, 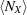, was fixed at 10 unit cells. Each generated set involved a total of 2000 nanocrystals. The orientations of simulated crystals were constrained to diffract into a selected crystallographic plane, the (*hk*0) plane. A significant increase in the total number of crystals would be required to fill a complete three-dimensional diffraction volume.

Within each simulated set of crystals, individual diffraction patterns were calculated for each crystal on a 1024 × 1024 array [see Dilanian *et al.* (2013 ▸) for more details] using the published atomic positions and Debye–Waller factors (PDB entry 4AK8). The crystal set corresponding to the smallest average size was also calculated on a 340 × 340 pixel array. Knowledge of the crystal structure, such as the unit-cell parameters and symmetry operations, was employed during the calculation of individual diffraction patterns and the subsequent merging. All diffraction patterns were calculated in the (*hk*0) crystallographic plane to 5.0 Å resolution. Additional crystal disorder effects were not included in this simulation study.

Merging of the individual diffraction patterns was performed for each collection of crystals to create seven two-dimensional diffraction patterns defined by varying mean crystal dimensions. Since we are only considering the two-dimensional diffraction patterns in the (*hk*0) crystallographic plane, the dimension of the nanocrystals along the [001] direction is not relevant in this case. The proposed approach, however, can be applied to any arbitrarily oriented reciprocal-lattice plane and extended to the fitting of three-dimensional diffraction data.

### 4.2. Analysis   

The accurate and robust extraction of structure-factor moduli is the primary objective of the presented whole-pattern fitting technique. Molecular structural information is encoded within these values and the structural analysis of protein crystals for diminishing crystal size requires suitable methods with which to read out such structural information. Structure-factor moduli estimated from whole-pattern fitting, 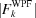, are compared here both to the values, 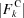, calculated from published structural data (PDB entry 4AK8) and also to the values, 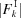, found from the integration approach. The respective relative errors, 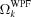 = 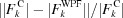 and 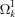 = 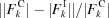, of the extracted structure-factor moduli are assessed for both the whole-pattern fitting and the integration methods. It is shown here that the accuracy of extracted structure-factor moduli *via* the integration of merged diffraction data is sensitive to the chosen integration area, whereas the values found from the presented whole-pattern fitting approach do not require integration and exhibit improved accuracy for diffraction data merged from small average crystal sizes.

**Figure 1:**
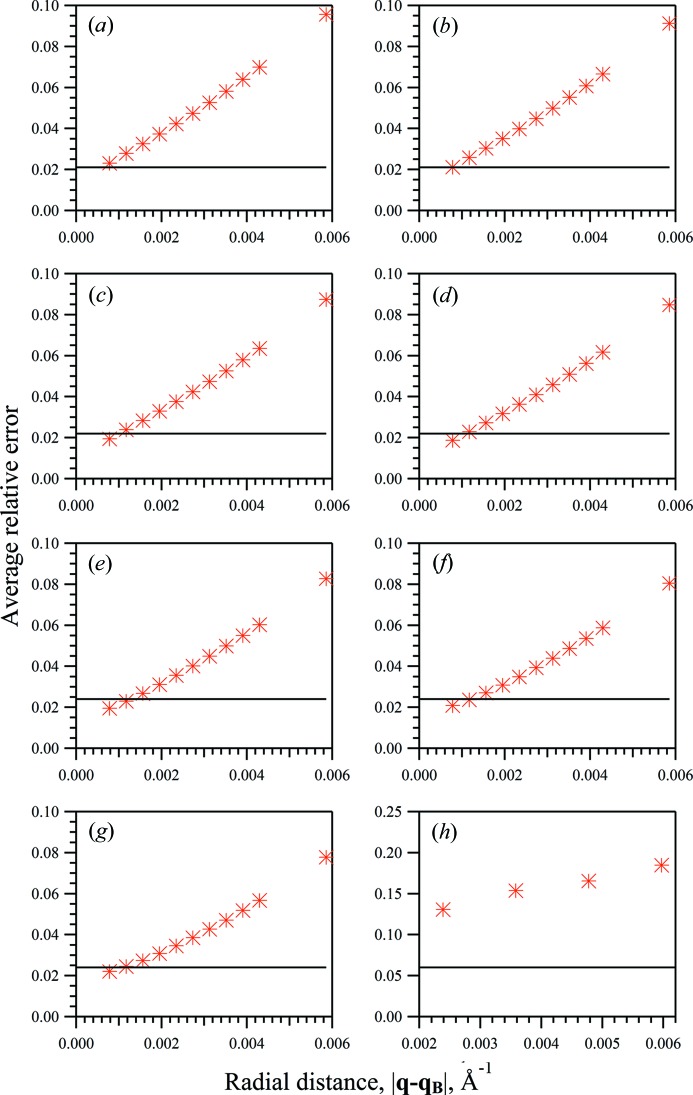
Structure-factor moduli average relative errors from the two-dimensional whole-pattern fitting approach (line) and from the integration approach (* symbol) against integration area for merged diffraction patterns from sets of 2000 crystallites with varying mean unit-cell dimensions along the *Y* direction, 〈*N_Y_*〉, and with a mean of 10 unit cells along the *X* direction, 〈*N_X_*〉: (*a*) 〈*N_Y_*〉 = 30, (*b*) 〈*N_Y_*〉 = 35, (*c*) 〈*N_Y_*〉 = 40, (*d*) 〈*N_Y_*〉 = 45, (*e*) 〈*N_Y_*〉 = 50, (*f*) 〈*N_Y_*〉 = 55, (*g*) 〈*N_Y_*〉 = 60, (*h*) 〈*N_Y_*〉 = 30. (*a*)–(*g*) The diffraction patterns were simulated on a 1024 × 1024 pixel array to 5 Å resolution. (*h*) The diffraction pattern was simulated on a 340 × 340 pixel array to 5 Å resolution. The integration area was defined by a circle with radius varied in the range 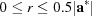.

In this investigation, a variety of integration areas were trialled for the extraction of structure-factor moduli *via* integration for the test diffraction data from the protein langerin. Scaling and thermal correction factors were estimated and applied for all sets of structure-factor moduli found from each integration area. This was performed using Wilson statistics (Giacovazzo, 2011 ▸), as with the structure-factor moduli extracted *via* whole-pattern fitting. The average relative error, 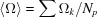, was assessed, where 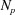 is the number of extracted structure-factor moduli. Figs. 1 ▸(*a*)–1 ▸(*g*) show the average relative errors from integration for the seven merged diffraction patterns for a variety of integration areas. The integration area was defined by a circle with the radius, 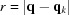, varied from zero (the Bragg position) to half of the distance between the nearest Bragg reflection, 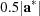, where 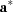 is the reciprocal-lattice parameter, which corresponds, in our case, to the boundary of the Wigner–Seitz cell along the [100] (or [010]) direction of the reciprocal lattice. The average error found from whole-pattern fitting is also indicated in Figs. 1 ▸(*a*)–1 ▸(*g*). These values are represented by solid lines given that the extracted structure-factor moduli are independent of integration area with this technique. It should be noted that in each instance and in both the whole-pattern fitting and the integration methods, a fixed set of several very weak reflections has been excluded from the assessment of average relative errors. These were excluded due to the weighted errors being considerably higher in these cases and thus having a significant impact on the overall averages. The problem of weak reflections will be discussed later in this section.

The whole-pattern fitting approach is shown in Fig. 1 ▸ to improve the accuracy of extracted structure-factor moduli for the smallest average crystal sizes. This is shown in Figs. 1 ▸(*a*)–1 ▸(*b*) and, most significantly, in Fig. 1 ▸(*h*), for which the smallest average crystal size is represented with fewer pixels simulated. Similar accuracy is achieved by both approaches for larger average crystal sizes, shown in Figs. 1 ▸(*c*)–1 ▸(*g*). The relative success of the integration method shown in Figs. 1 ▸(*a*)–1 ▸(*g*) can be understood from the size of the integration areas: the smallest average errors were found from the smallest integration lengths. It follows that, as the integration length is decreased, the extracted values approach the values of the heights of the Bragg reflections. Given that additional sources of structural disorder were not included in the simulated diffraction data, it is to be expected that the peak heights of well resolved, high-signal Bragg reflections would provide accurate structure-factor moduli. However, both the number of pixels between adjacent Bragg positions and the signal-to-noise ratio of the diffraction data would often be lower for experimentally collected SFX data. The average errors found *via* integration indicate that intensity contributions from neighbouring peaks can quickly begin to contribute as the integration area is expanded. The accuracy of the whole-pattern fitting approach does not exhibit this sensitivity.

The whole-pattern fitting approach allows peak contributions to extend continuously throughout the modelled diffracted intensity distribution. Limited integration or calculation areas are not required for the extraction of structure-factor moduli, as outlined earlier. As in powder diffraction Le Bail analysis, the whole-pattern fitting approach allows for individual reflections to be isolated and untangled from the contributions of nearby Bragg reflections. Average relative errors found [Figs. 1 ▸(*a*)–1 ▸(*g*)] indicate the stability of structure-factor moduli extracted by the whole-pattern fitting formulation. This approach is shown to produce an accuracy comparable to the integration method using optimal integration areas, while the results from integration decrease in quality for varying integration area. Improved accuracy from whole-pattern fitting for diffraction data merged from the smallest average crystal sizes is also evident. This favours the use of the whole-pattern fitting approach particularly for the analysis of data from small average crystal sizes in SFX experiments, with stable results found that are independent of calculation areas.

An average of slightly over 30 pixels was held between the positions of neighbouring Bragg reflections due to the large number of pixels used in this simulation study. This provided the capacity for detailed distributions of peak shapes to test the accuracy of the extended pseudo-Voigt function and of the whole-pattern fitting approach. However, it is acknowledged that these pixel numbers are not necessarily realistic in the case of the analysis of high-resolution SFX experimental data. To test this, simulations were performed of the diffraction pattern on a 340 × 340 pixel array to 5.0 Å resolution for a collection of 2000 nanoscale crystals of the test protein with mean dimensions of 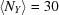 and 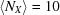 unit cells along orthogonal real-space dimensions. In this case, as few as 9–10 pixels exist from Bragg peak to Bragg peak. Both the whole-pattern fitting and the integration approaches were used to extract structure-factor moduli. A selection of extracted values and corresponding relative errors, 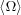, is provided in Table S1 (in the supporting information). Within Table S1, several weak reflections have been highlighted that have been excluded from the calculated average error, 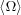. The structure-factor moduli values shown in Table S1 are provided with the optimal integration area for the integration method. The dependence of the average relative error upon these areas was also tested and is shown in Fig. 1 ▸(*h*). It is apparent that the whole-pattern fitting approach was able to extract structure-factor moduli to an accuracy similar to the earlier and more pixel-dense case. Accuracy was significantly diminished for the structure-factor moduli extracted *via* integration, however. The dependence on integration area was also further exacerbated. This can be expected due to the fineness of integration area choices being limited by the fewer number of pixels present. It is worth noting that the ability of the integration method to approach the peak-height values of the Bragg reflections is hindered by the lower density of pixels and that the accuracy of extracted structure-factor moduli is poorly affected as a result. The whole-pattern fitting approach does not appear to exhibit the same sensitivity. In cases where the peak heights of Bragg reflections cannot be expected to be reliably extracted for structure-factor moduli estimation, whole-pattern fitting techniques may be valuable.

### 4.3. Peak-shape parameters   

**Figure 2:**
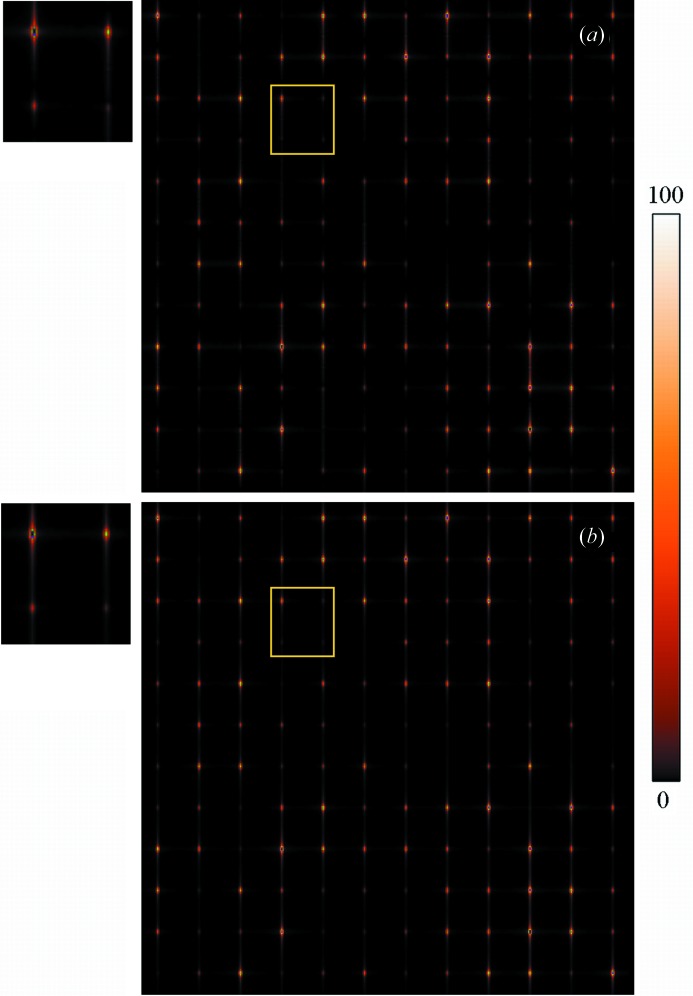
Merged two-dimensional diffraction patterns, (*a*) simulated and (*b*) fitted using the whole-pattern fitting approach, in the crystallographic (*hk*0) plane from a stream of 2000 crystals of various sizes with mean dimensions of 10 and 60 unit cells along the *X* and *Y* directions, respectively. Only one quadrant of the diffraction pattern is shown.

**Figure 3:**
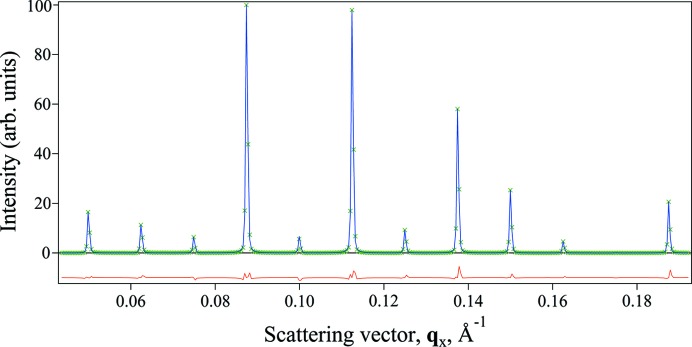
Cross sections through Bragg reflections along the (*h*, 14, 0) crystallographic direction through the simulated (green crosses) and fitted (blue lines) diffracted intensity distributions. The residual difference between the simulated and fitted distributions is shown (red line, offset below the positive intensity axis for clarity). The simulated diffracted intensity distribution was formed by the merging of patterns from 2000 crystals of mean unit-cell dimensions of 10 and 30 along the *X* and *Y* directions, respectively. Intensity values have been scaled to below 100 arbitrary units.

Peak-shape parameters found from the fitting procedures are given in Table 1 ▸ for each of the simulated diffraction patterns. No prior knowledge of the protein structure was used in fitting procedures, excluding the unit-cell parameters for the estimation of accurate Bragg peak positions. Fitting procedures were restricted to a single quadrant of the (*hk*0) crystallographic plane given that, in this case, the tetragonal symmetry of the test protein structure can be exploited. Selected sections in the (*hk*0) crystallographic plane of the simulated and fitted diffraction patterns are shown in Fig. 2 ▸ for the sets of generated crystallites with the largest average unit-cell dimension, 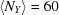. An example of cross sections of the simulated and fitted diffracted intensity distributions is shown in Fig. 3 ▸. Fig. 3 ▸ contains cross sections of the simulated and fitted intensity distributions for the smallest average unit-cell case, 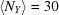, through Bragg reflections along the (*h*, 14, 0) crystallographic direction. Fitting of the simulated diffraction patterns was achieved with *R* factors of 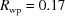, 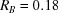 and 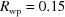, 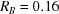 for the cases with the smallest, 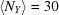, and the largest, 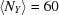, average unit-cell dimensions, respectively.

The set of mixing parameters, 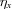, obtained from the fitting procedures, Table 1 ▸, demonstrates the dependence of the character of the peak-shape description upon the average size of the collection of crystallites. Put differently, diffraction patterns simulated with smaller average crystal sizes obtained smaller average mixing parameters during fitting procedures. This indicates a trend of greater contribution from the Lorentzian component in the fitted pseudo-Voigt functions for diffraction data merged from crystals of smaller average size. This might be interpreted as indicating more diffuse scattering in these cases, with less localized distributions of intensities found to be fitted around Bragg reflections.

Throughout the fitting process, inversion of the ***P*** matrix was employed to estimate structure-factor moduli according to current peak-shape parameters. The condition number can be estimated by the ratio of the largest to the smallest singular value of the ***P*** matrix to test whether the system of linear equations, equation (7), is well conditioned. Condition numbers calculated from final peak-shape parameters were found to be in the range of 1.1–1.2, indicating that the formulation used was well conditioned in the cases considered here, allowing the simple Gaussian elimination method to be used for matrix inversion.

### 4.4. ‘Overlapping’ Bragg peak contributions   

Each individual term in the summation of equation (5) represents the intensity distribution for a given Bragg reflection untangled from the nearby Bragg reflections. The intensity distribution around a given Bragg reflection in the merged diffraction pattern can be represented by
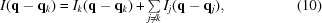
where 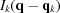 is the modelled intensity distribution for the *k*th peak and 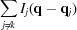 is the combined contribution of other Bragg peaks around the Bragg position, 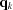. This second term defines the contribution of surrounding Bragg reflections to a given Bragg reflection in our modelled intensity distribution. The magnitude of this term determines the extent to which the Bragg reflections can be considered as isolated. The combined intensity distribution, 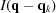, is used to model the total intensity distribution from the *k*th peak during whole-pattern fitting.

**Figure 4:**
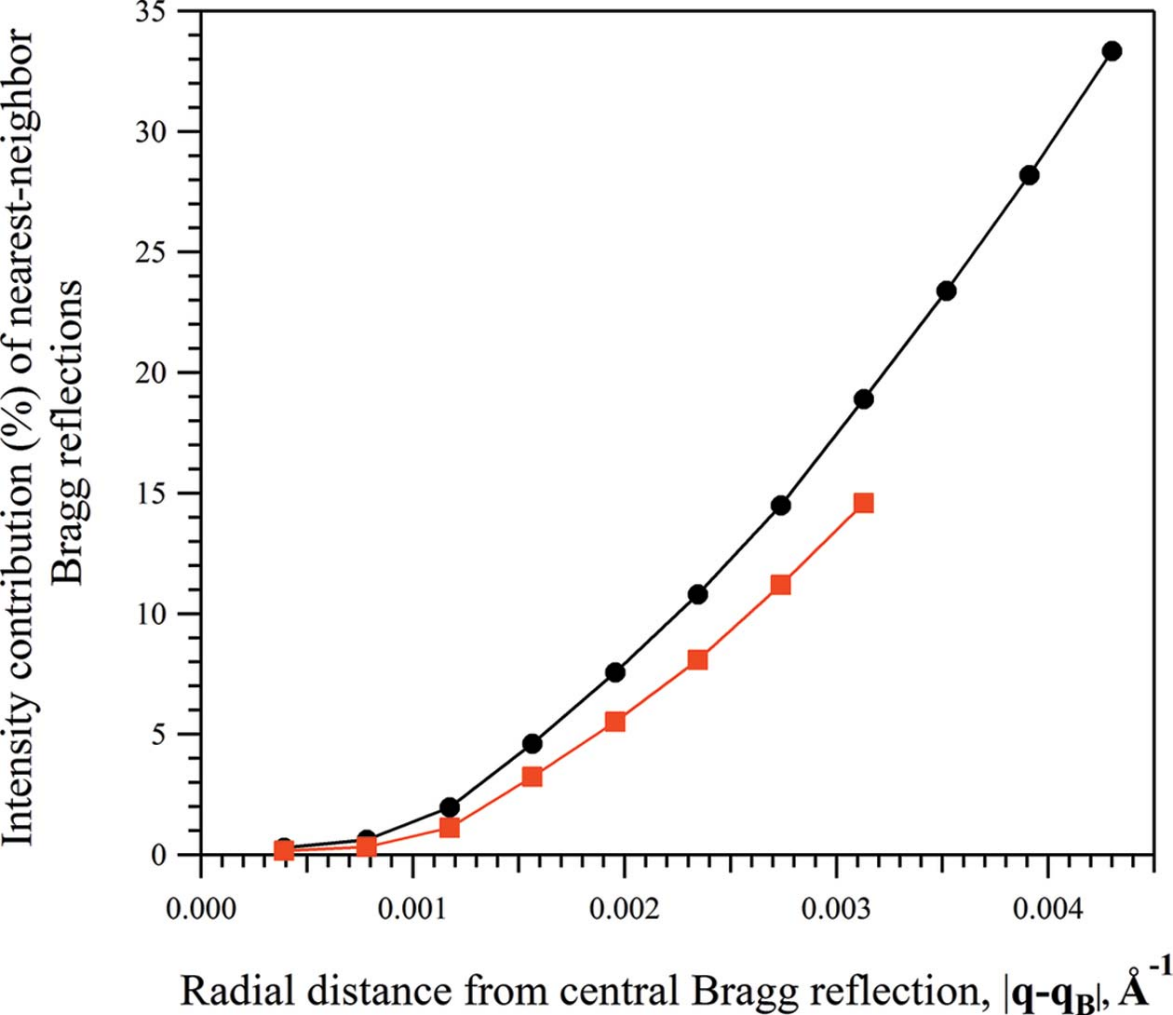
The percentage contribution of nearest-neighbour Bragg reflections to the total modelled intensity distribution of the (980) Bragg reflection [|*F* ^WPF^(980)| = 555.6, |*F* ^C^(980)| = 551.1]. The percentage contribution was calculated as the percentage of the intensity distribution modelled by the surrounding Bragg reflections [the second term of equation (10)] relative to the total intensity modelled at radial distances from the Bragg position of the (980) reflection. The analysis was performed on two merged diffraction patterns generated from a collection of 2000 nanoscale crystals of the test protein with different mean unit-cell dimensions along the *Y* direction and with a mean of 10 unit cells along the *X* direction, 〈*N_X_*〉 = 10: (black, circles) 〈*N_Y_*〉 = 30, (red, squares) 〈*N_Y_*〉 = 60.

In order to evaluate the influence of the nearest-neighbour reflections on the intensity distribution of the Bragg reflection, we calculated the first and the second terms of equation (10) using the peak-shape parameters obtained *via* whole-pattern fitting. The contribution of two terms was calculated for various radial distances, 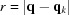, from the Bragg position of a selected reflection. Similarly to the previous analysis (Fig. 1 ▸), the radial distance varied in the range 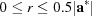. Only the intensities exceeding 2% of the peak intensity of the corresponding Bragg reflection were considered in the calculations. The analysis was performed on two merged diffraction patterns generated from a collection of 2000 nanoscale crystals of the test protein with mean dimensions of 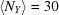, 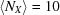, and 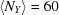, 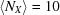, respectively. As one can see from Fig. 4 ▸, the contribution of the second term increases with the distance from the Bragg position of the selected reflection, quickly reaching 10% (or more) of the total intensity. The obtained results are in a good agreement with results shown in Fig. 1 ▸, in that the wider the integration area, the stronger the influence of the nearest Bragg reflections on the intensity distribution of the selected reflection and, consequently, the bigger the error in the determination of the corresponding integrated intensity. The contribution of the second term of equation (10) to the total intensity of the Bragg reflection can differ for different reflections. In particular, the influence is negligible when a strong Bragg reflection is surrounded by weak reflections. Conversely, the intensity distribution of a weak Bragg reflection surrounded by very strong reflections will largely be determined by the contributions of surrounding reflections.

This may be the cause of some difficulties present in both of the extraction methods considered here, which make the accurate estimation of structure-factor moduli for several weak reflections problematic. Further development of the whole-pattern fitting method may be required for the treatment of weak reflections, together with the use of more robust and numerically stable algorithms for less well conditioned ***P*** matrices. In the current approach, all Bragg reflections are fitted simultaneously with either common peak-shape parameters or common dependences of the peak-shape parameters on the scattering vector. The weak reflections that were poorly estimated were often surrounded by much stronger reflections; this may have affected the accuracy with which these sets of reflections could be modelled with this approach. Further extensions could be made by performing the individual fitting of small numbers of selected weak reflections, following whole-pattern fitting procedures. A similar approach is present in powder diffraction analysis, known as partial profile relaxation (Izumi, 2003 ▸). This would be expected to increase the accuracy of structure-factor moduli for weak reflections by allowing those with nearby strong reflections to have independent peak-shape parameters.

## 5. Conclusion   

Presented here is an approach that builds upon an established analysis technique in powder diffraction, the whole-pattern fitting method (Le Bail *et al.*, 1988 ▸). The impetus for this can be seen in the similarities between powder diffraction and merged SFX data sets. In both instances, peak-shape distributions are formed by the shape, size and disorder characteristics of a large set of independent scatterers. It is shown here that appropriate extension of the whole-pattern fitting technique can be used to closely model SFX diffraction patterns and to extract integrated intensity information of Bragg reflections. This follows the work of Dilanian *et al.* (2013 ▸) in the use of a continuous description of the diffraction pattern obtained from a distribution of protein nanocrystals.

This analysis indicates that the whole-pattern fitting method is a feasible approach for the extraction of intensity information from SFX data. Parameters obtained from fitting procedures show some dependence on the average size of contributing crystals. Flexibility is provided in the form of peak-shape distributions to fit merged diffraction patterns from crystals of varying mean sizes using smooth analytical functions adopted from the analysis of powder diffraction data. Further improvements in this approach might be made with closer consideration of the theoretical basis of the average shape-transform distributions formed by collections of nanocrystals.

The strength of the whole-pattern fitting approach in isolating Bragg reflections is particularly desirable in cases where substantial inter-Bragg scattering occurs – such as when unit-cell parameters are large yet the dimensions of the contributing crystals are small. This supports the application of the whole-pattern fitting approach in the analysis of high-resolution experimental SFX data from large collections of protein nanocrystals.

Several important aspects of the SFX approach were outside the scope of the analysis presented in this article. We have analysed the defect-free structure of the protein crystal. This allowed all peaks to be fitted with the same 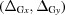, 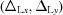 parameters. In general, each of the adjustable parameters 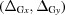 and 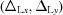 can be represented by an analytical function of the scattering vector, **q**, to take into account effects of the structural disorder on the diffraction pattern of protein nanocrystals. The unit-cell parameters may also vary from crystal to crystal, or even within one crystal. This will lead to a shift of Bragg reflections from ideal positions. All of these problems are well known in single-crystal and powder diffraction crystallography (*e.g.* Ungár & Gubicza, 2007 ▸; Palosz *et al.*, 2003 ▸) and can further be incorporated into the analysis of SFX data. In our analysis we considered all the molecules which form incomplete unit cells on the surface of the protein crystals as independently scattered objects. In this case, molecules from incomplete unit cells contribute only to the background scattering (Welberry, 2004 ▸). This assumption is incorrect, however, for extremely small crystals, comprised of several molecular clusters, when the influence of such molecules on the diffraction pattern is strong (Chen & Millane, 2013 ▸). It should also be noted that the definitions of ‘crystal lattice’ and the ‘unit cell’ are not entirely clear in this case and a separate analysis of this situation is required.

## Supplementary Material

Supplementary section containing structure-factor moduli extracted via whole-pattern fitting and integration. DOI: 10.1107/S2052252516001238/cw5009sup1.pdf

## Figures and Tables

**Table 1:** Merged diffraction pattern adjustable parameters from size-varying crystallite sets

Adjustable parameters	Mean unit-cell dimensions, 〈*N_Y_*〉, in log-normal distribution for 2000 crystallites						
	30	35	40	45	50	55	60
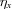	0.594	0.543	0.540	0.525	0.524	0.536	0.517
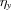	0.465	0.502	0.650	0.736	0.760	0.842	0.876
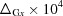 , Å^−1^	5.78	5.85	5.85	5.86	5.99	5.94	5.96
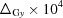 , Å^−1^	3.36	3.20	3.08	3.07	2.94	3.01	2.89
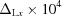 , Å^−1^	6.20	6.178	5.434	5.23	4.91	4.89	4.83
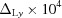 , Å^−1^	1.98	1.96	1.91	1.69	1.52	1.33	1.28

## Appendix A The scattering factor for a finite crystal

The scattering factor for a nanocrystal is often expressed as the product of a finite lattice transform and unit-cell scattering factor. Here we describe the alternative expression used throughout this work [equations (1) and (15)] and show its equivalence to the former formulation [equation (16)] for the ideal case of a finite crystal composed solely of whole, identically ordered unit cells. This should be viewed simply as an example presented for an idealized case. Discrepancies in the scattering factor formulations can be expected to arise for ensembles of real crystals for which the diffracted intensity distributions will be shaped by variations in factors such as size, shape, disorder and strain.

The formulation of the diffracted X-ray intensity from a finite crystal is briefly reviewed here. Following representations of Patterson (1939 ▸) and Ewald (1940 ▸), the electron density of a finite crystal, , can be defined as

where **r** is a vector in real space and  is the electron density of an effectively infinite crystal lattice, such as in macroscopic crystals used in conventional crystallography. The shape of the crystal is denoted  and can be written as

Here, *V*_C_ is the volume of the crystal and determines its boundary. We adopt the further definition of Guinier (1963 ▸) that  is bounded such that whole unit cells are contained within the crystal volume. The scattering amplitude, *F*_C_(**q**), can then be represented as

where  is the scattering amplitude from an effectively infinite crystal and  is the Fourier transform of . Ignoring nuclear motion, the scattering amplitude from an infinite crystal can be expressed as

where  is the scattering factor of the unit cell sampled at the Bragg positions, , with the Dirac delta function distribution, . This provides the scattering factor for the finite crystal as

which is identical to the form of the scattering factor provided in equation (1). Recent work (*e.g.* Kirian *et al.*, 2010 ▸, 2014 ▸; Spence *et al.*, 2011 ▸) has used a formulation of the scattering factor for a nanocrystal that can be expressed as

where  is the continuous scattering factor of the unit cell and  is the Fourier transform of the lattice function of the nanocrystal, . The lattice function in real space can be written as a finite series of Dirac delta functions located at the corner of each occupied unit cell:

where *N* denotes the number of unit cells in the nanocrystal. We seek to demonstrate here that both formulations [*i.e.* equations (15) and (16)] are equivalent for the ideal case considered in this appendix.

The scattering factor of the unit cell, , is a band-limited function and thus can be reproduced with a minimal sampling rate given by the Shannon–Nyquist sampling, which is given here by the Bragg locations. The scattering factor for the unit cell can consequently be expressed as

where  is the Fourier transform for the shape function of the unit cell centred on the Bragg locations, . The unit-cell shape function can be defined as

where  is the volume of the unit cell and defines its boundary. Knowledge of  at the Shannon–Nyquist sampling points, , is sufficient to interpolate to find  at other positions using the unit-cell shape transform, .

The scattering factor for the nanocrystal given in equation (16) can then be expressed as

The product, , can be written as

In our approach, the following relationship is used:

Equations (21) and (22) are not equal for general expressions due to convolution not holding associativity properties with multiplication. These equations are equivalent in this work, however, due to the characteristics of the particular functions considered here. This can be shown by considering the inverse Fourier transform of each expression. The inverse Fourier transform of equation (21) is given by

where  indicates the inverse Fourier transform operation and *K* is an integer given by the dot product of a lattice point vector, , and a reciprocal-lattice point vector, . The reciprocal relationship of the points  and  arises from the band-limited nature of the scattering factor of the unit cell, . Given that *K* is an integer, the phase factor  is equal to one. This provides the inverse Fourier transform of equation (21) as

This is equal to the inverse Fourier transform of equation (22), given the following:

The equality of equations (24) and (25) arises from the characteristics of the lattice function, , and from the sampling points in reciprocal space, . These characteristics allow the convolution operator to be applied equivalently in equations (21) and (22). The order of the application of convolution operations between shape functions and unit-cell electron-density distributions can create differences in models for the scattering factor for a nanocrystal when the presence of incomplete unit cells is considered (Ino & Minami, 1979 ▸; Beyerlein, 2011 ▸). Alternative scattering factor models have been evaluated in powder diffraction studies (Ino & Minami, 1984 ▸); further consideration of the influence of crystal boundary effects in ensembles of finite crystals may be required in future work. The product, , can then be shown to be equivalent to the Fourier transform of the shape function of the crystal centred at the Bragg positions, , given that

where  indicates the Fourier transform operation and the presence of whole unit cells has been assumed. Substitution into equation (20) then provides the result,

showing the equivalence of the models for the scattering factor of a nanocrystal for the cases we consider here.

A consequence of this is that it is implicitly assumed that the sampling rate for the scattering factor for the unit cell, , is the Shannon–Nyquist sampling rate when considering ‘overlapping’ Bragg peak distributions in this paper. The spreading of Bragg peak distributions can also be understood as incorporating the interpolation of  to locations in between these sampling points, *i.e.* interpolation between the Bragg locations.

## References

[bb1] Aquila, A. *et al.* (2012). *Opt. Express*, **20**, 2706–2716. 10.1364/OE.20.002706 PMC3413412 22330507

[bb2] Barty, A., Kirian, R. A., Maia, F. R. N. C., Hantke, M., Yoon, C. H., White, T. A. & Chapman, H. (2014). *J. Appl. Cryst.* **47**, 1118–1131. 10.1107/S1600576714007626 PMC4038800 24904246

[bb3] Beyerlein, K. R. (2011). *Simulation and modeling of the powder diffraction pattern from nanoparticles: studying the influence of surface strain*. PhD thesis, Georgia Institute of Technology, USA. Retrieved from https://smartech.gatech.edu/handle/1853/41211.

[bb4] Boutet, S. *et al.* (2012). *Science*, **337**, 362–364.

[bb5] Caylor, C. L., Dobrianov, I., Lemay, S. G., Kimmer, C., Kriminski, S., Finkelstein, K. D., Zipfel, W., Webb, W. W., Thomas, B. R., Chernov, A. A. & Thorne, R. E. (1999). *Proteins*, **36**, 270–281. 10409821

[bb6] Chabrol, E., Thépaut, M., Dezutter-Dambuyant, C., Vivès, C., Marcoux, J., Kahn, R., Valladeau-Guilemond, J., Vachette, P., Durand, D. & Fieschi, F. (2015). *Biophys. J.* **108**, 666–677. 10.1016/j.bpj.2014.10.075 PMC4317532 25650933

[bb7] Chapman, H. N. *et al.* (2011). *Nature (London)*, **470**, 73–77.

[bb8] Chen, J. P. J. & Millane, R. P. (2013). *J. Opt. Soc. Am. A*, **30**, 2627–2634. 10.1364/JOSAA.30.002627 24323025

[bb9] Demirci, H. *et al.* (2013). *Acta Cryst.* F**69**, 1066–1069.

[bb10] Dilanian, R. A., Streltsov, V. A., Quiney, H. M. & Nugent, K. A. (2013). *Acta Cryst.* A**69**, 108–118. 10.1107/S0108767312042535 23250067

[bb11] Elser, V. (2013). *Acta Cryst.* A**69**, 559–569. 10.1107/S0108767313023362 24132217

[bb12] Ewald, P. P. (1940). *Proc. Phys. Soc.* **52**, 167–174.

[bb13] Feher, G. & Kam, Z. (1985). *Methods Enzymol.* **114**, 77–112. 10.1016/0076-6879(85)14006-1 4079780

[bb14] Giacovazzo, C. (2011). Editor. *Fundamentals of Crystallography*, 3rd ed. Oxford University Press.

[bb15] Grant, M. L. & Saville, D. A. (1994). *J. Phys. Chem.* **98**, 10358–10367.

[bb16] Guinier, A. (1963). *X-ray Diffraction in Crystals, Imperfect Crystals, and Amorphous Bodies*. San Francisco: W. H. Freeman and Company.

[bb17] Hill, R. J. & Fischer, R. X. (1990). *J. Appl. Cryst.* **23**, 462–468.

[bb18] Hosemann, R. & Bagchi, S. N. (1962). *Direct Analysis of Diffraction by Matter*. Amsterdam: North-Holland Publishing Company.

[bb19] Ino, T. & Minami, N. (1979). *Acta Cryst.* A**35**, 163–170.

[bb20] Ino, T. & Minami, N. (1984). *Acta Cryst.* A**40**, 538–544.

[bb21] Izumi, F. (2003). *J. Ceram. Soc. Jpn*, **111**, 617–623.

[bb22] James, R. W. (1954). *The Crystalline State*, Vol. II, *The Optical Principles of the Diffraction of X-rays*. London: G. Bell and Sons.

[bb24] Johansson, L. C. *et al.* (2012). *Nat. Methods*, **9**, 263–265. 10.1038/nmeth.1867 PMC3438231 22286383

[bb23] Johansson, L. C. *et al.* (2013). *Nat. Commun.* **4**, 2911. 10.1038/ncomms3911 PMC3905732 24352554

[bb25] Kabsch, W. (2010). *Acta Cryst.* D**66**, 125–132. 10.1107/S0907444909047337 PMC2815665 20124692

[bb26] Kabsch, W. (2014). *Acta Cryst.* D**70**, 2204–2216. 10.1107/S1399004714013534 PMC4118830 25084339

[bb27] Kirian, R. A., Bean, R. J., Beyerlein, K. R., Barthelmess, M., Yoon, C. H., Wang, F., Capotondi, F., Pedersoli, E., Barty, A. & Chapman, H. N. (2015). *Phys. Rev. X*, **5**, 011015.

[bb28] Kirian, R. A., Bean, R. J., Beyerlein, K. R., Yefanov, O. M., White, T. A., Barty, A. & Chapman, H. N. (2014). *Philos. Trans. R. Soc. Lond. B Biol. Sci.* **369**, 20130331. 10.1098/rstb.2013.0331 PMC4052867 24914158

[bb30] Kirian, R. A., Wang, X., Weierstall, U., Schmidt, K. E., Spence, J. C. H., Hunter, M., Fromme, P., White, T., Chapman, H. N. & Holton, J. (2010). *Opt. Express*, **18**, 5713–5723. 10.1364/OE.18.005713 PMC4038330 20389587

[bb31] Kirian, R. A., White, T. A., Holton, J. M., Chapman, H. N., Fromme, P., Barty, A., Lomb, L., Aquila, A., Maia, F. R. N. C., Martin, A. V., Fromme, R., Wang, X., Hunter, M. S., Schmidt, K. E. & Spence, J. C. H. (2011). *Acta Cryst.* A**67**, 131–140. 10.1107/S0108767310050981 PMC3066792 21325716

[bb32] Koopmann, R. *et al.* (2012). *Nat. Methods*, **9**, 259–262. 10.1038/nmeth.1859 PMC3429599 22286384

[bb33] Kupitz, C. *et al.* (2014). *Nature (London)*, **513**, 261–265.

[bb34] Langford, J. I. (1999). *Defect and Microstructure Analysis by Diffraction*, edited by R. L. Snyder, J. Fiala & H.-J. Bunge, pp. 59–81. Oxford University Press.

[bb35] Le Bail, A., Duroy, H. & Fourquet, J. L. (1988). *Mater. Res. Bull.* **23**, 447–452.

[bb36] Leslie, A. G. W. & Powell, H. R. (2007). *Evolving Methods for Macromolecular Crystallography*. NATO Science Series, Vol. 245, pp. 45–51.

[bb37] Liu, W. *et al.* (2013). *Science*, **342**, 1521–1524.

[bb38] Liu, H., Zatsepin, N. A. & Spence, J. C. H. (2014). *IUCrJ*, **1**, 19–27. 10.1107/S2052252513025530 PMC4104966 25075316

[bb39] Malkin, A. J. & Thorne, R. E. (2004). *Methods*, **34**, 273–299. 10.1016/j.ymeth.2004.03.020 15325647

[bb40] Neutze, R., Wouts, R., van der Spoel, D., Weckert, E. & Hajdu, J. (2000). *Nature (London)*, **406**, 752–757. 10.1038/35021099 10963603

[bb41] Palosz, B., Grzanka, E., Gierlotka, S., Stel’makh, S., Pielaszek, R., Lojkowski, W., Bismayer, U., Neuefeind, J., Weber, H.-P. & Palosz, W. (2003). *Phase Transitions*, **76**, 171–185.

[bb42] Patterson, A. L. (1939). *Phys. Rev.* **56**, 972–977.

[bb43] Redecke, L. *et al.* (2013). *Science*, **339**, 227–230.

[bb44] Rietveld, H. M. (1967). *Acta Cryst.* **22**, 151–152.

[bb45] Spence, J. C. H., Kirian, R. A., Wang, X., Weierstall, U., Schmidt, K. E., White, T., Barty, A., Chapman, H. N., Marchesini, S. & Holton, J. (2011). *Opt. Express*, **19**, 2866–2873. 10.1364/OE.19.002866 21369108

[bb46] Spence, J. C. H., Weierstall, U. & Chapman, H. M. (2012). *Rep. Prog. Phys.* **75**, 102601. 10.1088/0034-4885/75/10/102601 22975810

[bb47] Suortti, P., Ahtee, M. & Unonius, L. (1979). *J. Appl. Cryst.* **12**, 365–369.

[bb48] Suortti, P. & Jennings, L. D. (1977). *Acta Cryst.* A**33**, 1012–1027.

[bb49] Ungár, T. & Gubicza, J. (2007). *Z. Kristallogr.* **222**, 114–128.

[bb50] Welberry, T. R. (2004). *Diffuse X-ray Scattering and Models of Disorder*. Oxford University Press.

[bb51] White, A. (2014). *Philos. Trans. R. Soc. Lond. B Biol. Sci.* **369**, 20130330. 10.1098/rstb.2013.0330 PMC4052866 24914157

[bb52] White, T. A., Kirian, R. A., Martin, A. V., Aquila, A., Nass, K., Barty, A. & Chapman, H. N. (2012). *J. Appl. Cryst.* **45**, 335–341.

[bb54] Yefanov, O., Gati, C., Bourenkov, G., Kirian, R. A., White, T. A., Spence, J. C. H., Chapman, H. N. & Barty, A. (2014). *Philos. Trans. R. Soc. Lond. B Biol. Sci.* **369**, 20130333. 10.1098/rstb.2013.0333 PMC4052869 24914160

[bb55] Young, R. A. & Wiles, D. B. (1982). *J. Appl. Cryst.* **15**, 430–438.

