# Supplementary material for: Whole-pattern fitting technique in serial femtosecond nanocrystallography
Source: IUCrJ. 2016 Feb 12;3(Pt 2):127–38. doi: 10.1107/S2052252516001238 (PMC4775161; doi:10.1107/S2052252516001238)
Supplement: Supplementary file 1 [file m-03-00127-sup1.pdf]

IUCrJ

Volume 3 (2016)

Supporting information for article:

Whole-pattern fitting technique in serial femtosecond nanocrystallography

Ruben A. Dilanian, Sophie R. Williams, Andrew V. Martin, Victor A. Stretsov and Harry M. Quiney

Whole-pattern fitting technique in serial femtosecond nanocrystallography

Ruben A. Dilanian, Sophie R. Williams, Andrew V. Martin, Victor A. Stretsov and Harry M. Quiney

Supplementary Information

Table S1. Structure factor moduli of selected Bragg reflections extracted from a merged diffraction pattern from the test protein using the whole-pattern fitting approach, $\left|F^{WPF}\right|$, the integration approach, $\left|F^{I}\right|$, and calculated using published data (PDB ID: 4AK8), $\left|F^{C}\right|$. Ω^{WPF} and Ω^{I} are the relative errors for $\left|F^{WPF}\right|$ and $\left|F^{I}\right|$ respectively. The diffraction pattern was calculated in the (*hk0*) crystallographic plane from a set of 2000 crystallites with a mean of 30 unit-cells along [100] direction and of 10 unit-cells along the [010] direction. The diffraction pattern was simulated on a 340x340 pixel array to 5.0 Å resolution. The average relative errors, $\left\langle\Omega^{WPF}\right\rangle$ = 0.06 and $\left\langle\Omega^{I}\right\rangle$ = 0.13, have been calculated with several weak reflections excluded (indicated in bold).

h	*k*	*l*	$\|F^C\|$	$\|F^{WPF}\|$	Ω^{WPF}	$\|F^I\|$	Ω^I	*h*	*k*	*l*	$\|F^C\|$	$\|F^{WPF}\|$	Ω^{WPF}	$\|F^I\|$	Ω^I
15	15	0	1479.44	1387.84	0.06	1392.08	0.06	9	15	0	348.55	334.33	0.04	348.15	0.00
15	14	0	765.45	721.29	0.06	673.07	0.12	9	14	0	1691.03	1580.70	0.07	1653.20	0.02
15	13	0	719.08	667.83	0.07	719.89	0.00	9	13	0	601.14	559.13	0.07	618.65	0.03
15	12	0	119.05	118.77	0.00	61.28	0.49	9	12	0	427.85	413.24	0.03	469.07	0.10
15	11	0	341.29	321.21	0.06	297.45	0.13	9	11	0	718.64	673.14	0.06	739.90	0.03
15	10	0	140.19	137.59	0.02	183.96	0.31	9	10	0	112.06	106.48	0.05	106.82	0.05
15	9	0	175.08	171.20	0.02	147.19	0.16	9	9	0	164.33	152.49	0.07	184.38	0.12
15	8	0	165.56	164.25	0.01	152.63	0.08	9	8	0	552.65	521.81	0.06	458.84	0.17
15	7	0	1122.08	1033.00	0.08	1219.34	0.09	9	7	0	746.68	704.97	0.06	766.41	0.03
15	6	0	885.83	833.21	0.06	925.19	0.04	9	6	0	153.23	148.84	0.03	121.82	0.20
15	5	0	564.17	536.45	0.05	473.25	0.16	9	5	0	276.23	259.15	0.06	284.48	0.03

15	4	0	71.54	63.84	0.11	87.91	0.23	9	4	0	117.62	108.81	0.07	121.12	0.03
14	15	0	167.96	170.06	0.01	118.12	0.30	8	15	0	1812.42	1707.92	0.06	1217.05	0.33
**14**	**14**	**0**	**20.86**	**50.87**	**1.44**	**107.26**	**4.14**	8	14	0	407.59	413.67	0.01	379.02	0.07
14	13	0	499.89	478.94	0.04	460.92	0.08	8	13	0	385.09	366.66	0.05	287.63	0.25
14	12	0	292.23	269.80	0.08	300.99	0.03	8	12	0	398.74	367.24	0.08	301.99	0.24
14	11	0	606.21	580.16	0.04	525.56	0.13	8	11	0	722.81	683.22	0.05	518.26	0.28
14	10	0	646.32	601.57	0.07	669.65	0.04	8	10	0	85.76	83.17	0.03	78.79	0.08
14	9	0	860.07	820.86	0.05	787.53	0.08	**8**	**9**	**0**	**8.01**	**27.15**	**2.39**	**39.13**	**3.89**
14	8	0	358.53	332.21	0.07	324.87	0.09	8	8	0	896.86	851.94	0.05	589.38	0.34
14	7	0	681.36	668.68	0.02	576.98	0.15	8	7	0	556.19	524.18	0.06	419.52	0.25
14	6	0	106.9	112.57	0.05	84.99	0.20	8	6	0	726.95	686.99	0.05	550.99	0.24
14	5	0	126.26	132.99	0.05	90.37	0.28	8	5	0	801.94	759.75	0.05	588.94	0.27
14	4	0	268.05	263.09	0.02	235.68	0.12	8	4	0	350.58	335.85	0.04	235.91	0.33
13	15	0	485.27	462.25	0.05	405.44	0.16	7	15	0	218.99	234.03	0.07	173.69	0.21
13	14	0	356.15	340.18	0.04	302.92	0.15	7	14	0	1709.09	1606.52	0.06	1708.67	0.00
13	13	0	1149.61	1059.99	0.08	1160.89	0.01	7	13	0	1367.24	1285.66	0.06	1358.00	0.01
13	12	0	216.53	214.50	0.01	174.26	0.20	7	12	0	662.9	625.29	0.06	624.81	0.06
13	11	0	1223.02	1133.39	0.07	1196.68	0.02	7	11	0	1236.52	1145.95	0.07	1323.64	0.07
13	10	0	426.65	403.27	0.05	358.40	0.16	7	10	0	311.26	289.61	0.07	333.50	0.07
13	9	0	918.49	836.41	0.09	1002.50	0.09	7	9	0	378.71	356.56	0.06	351.76	0.07
13	8	0	93.16	97.20	0.04	86.90	0.07	7	8	0	1498.04	1390.75	0.07	1427.53	0.05
13	7	0	146.65	111.93	0.24	370.75	1.53	7	7	0	245.37	228.05	0.07	262.17	0.07
13	6	0	893	824.27	0.08	855.68	0.04	7	6	0	671.73	629.14	0.06	662.50	0.01
13	5	0	257.16	232.58	0.10	311.06	0.21	7	5	0	778.76	724.03	0.07	756.87	0.03

13	4	0	1261.17	1171.24	0.07	1320.11	0.05	7	4	0	1146.02	1054.02	0.08	916.75	0.20
12	15	0	52.75	57.86	0.10	49.83	0.06	6	15	0	940.32	863.88	0.08	1017.66	0.08
12	14	0	848.74	797.85	0.06	738.27	0.13	6	14	0	420.59	401.57	0.05	337.22	0.20
12	13	0	779.71	728.85	0.07	678.33	0.13	6	13	0	76.35	85.81	0.12	117.27	0.54
**12**	**12**	**0**	**71.84**	**99.87**	**0.39**	**175.15**	**1.44**	6	12	0	142.85	136.45	0.04	141.40	0.01
**12**	**11**	**0**	**33.66**	**54.62**	**0.62**	**110.16**	**2.27**	6	11	0	259.31	246.29	0.05	292.42	0.13
12	10	0	351.8	326.95	0.07	330.60	0.06	6	10	0	226.49	215.36	0.05	268.17	0.18
12	9	0	179.03	170.95	0.05	143.44	0.20	6	9	0	1036.46	962.12	0.07	1047.75	0.01
12	8	0	661.74	598.35	0.10	614.85	0.07	6	8	0	92.91	84.33	0.09	95.98	0.03
12	7	0	1959.28	1801.15	0.08	2000.03	0.02	6	7	0	1658.77	1514.36	0.09	1541.82	0.07
12	6	0	400.67	372.29	0.07	347.30	0.13	6	6	0	1572.69	1466.04	0.07	1701.61	0.08
12	5	0	1451.56	1331.97	0.08	1401.46	0.03	6	5	0	2078.15	1949.36	0.06	2144.43	0.03
12	4	0	117.12	104.85	0.10	136.19	0.16	6	4	0	1193.89	1118.89	0.06	1050.45	0.12
11	15	0	1274.6	1177.02	0.08	1135.88	0.11	5	15	0	641.56	595.28	0.07	642.92	0.00
11	14	0	1310.49	1197.69	0.09	1317.61	0.01	5	14	0	569.29	528.86	0.07	682.76	0.20
11	13	0	155.39	141.02	0.09	147.16	0.05	5	13	0	641.29	595.14	0.07	669.67	0.04
11	12	0	87.11	79.89	0.08	90.34	0.04	5	12	0	385.65	358.87	0.07	415.65	0.08
11	11	0	601.96	551.54	0.08	587.95	0.02	5	11	0	227.69	214.54	0.06	204.94	0.10
11	10	0	267.75	243.51	0.09	260.08	0.03	5	10	0	347.6	326.38	0.06	328.63	0.05
11	9	0	345.26	309.20	0.10	362.41	0.05	**5**	**9**	**0**	**20.71**	**57.53**	**1.78**	**114.41**	**4.52**
11	8	0	1239.06	1137.83	0.08	1057.75	0.15	5	8	0	1539.322	1422.43	0.08	1380.10	0.10
11	7	0	963.78	871.50	0.10	885.66	0.08	5	7	0	734.355	675.08	0.08	897.25	0.22
11	6	0	169.48	172.22	0.02	179.56	0.06	5	6	0	1276.226	1172.88	0.08	1542.77	0.21
**11**	**5**	**0**	**4.7**	**75.16**	**14.99**	**169.74**	**35.1**	5	5	0	1304.423	1220.63	0.06	1285.38	0.01

11	4	0	232.14	226.63	0.02	294.30	0. 27	5	4	0	219.441	215.97	0.02	283.23	0. 29
10	15	0	1312.66	1217.34	0.07	1198.69	0. 09	4	15	0	740.335	693.27	0.06	718.09	0. 03
10	14	0	523.55	476.59	0.09	555.52	0. 06	4	14	0	696.137	654.89	0.06	785.23	0. 13
10	13	0	937.45	871.30	0.07	838.97	0. 11	4	13	0	316.475	298.97	0.06	332.46	0. 05
10	12	0	27.12	26.18	0.03	44.19	0. 63	4	12	0	248.249	238.62	0.04	183.95	0. 26
10	11	0	266.22	251.80	0.05	227.62	0. 14	4	11	0	141.391	137.01	0.03	90.99	0. 36
**10**	**10**	**0**	**44.03**	**96.23**	**1.19**	**176.72**	**3. 01**	**4**	**10**	**0**	**9.473**	**12.56**	**0.33**	**27.53**	**1. 91**
10	9	0	815.12	743.33	0.09	874.84	0. 07	4	9	0	132.585	128.05	0.03	107.41	0. 19
10	8	0	257.22	260.94	0.01	257.20	0. 00	4	8	0	611.249	579.59	0.05	503.28	0. 18
10	7	0	641.8	595.20	0.07	700.55	0. 09	4	7	0	206.147	190.97	0.07	320.08	0. 55
10	6	0	248.37	233.29	0.06	265.64	0. 07	4	6	0	437.267	410.74	0.06	591.85	0. 35
10	5	0	119.71	111.89	0.07	215.90	0. 80	4	5	0	346.362	328.70	0.05	346.36	0. 00
10	4	0	617.62	576.90	0.07	650.90	0. 05	4	4	0	1473.183	1384.90	0.06	1313.24	0. 11
